# Impacts of elevated CO_2_ and partial defoliation on mineral element composition in rice

**DOI:** 10.3389/fpls.2024.1450893

**Published:** 2024-11-18

**Authors:** Bo Gao, Shaowu Hu, Mingyuan Zhou, Liquan Jing, Yunxia Wang, Jianguo Zhu, Xingxing Sun, Kai Wang, Yulong Wang, Lianxin Yang

**Affiliations:** ^1^ Key Laboratory of Crop Genetics and Physiology of Jiangsu Province/Co-Innovation Center for Modern Production Technology of Grain Crops of Jiangsu Province, Yangzhou University, Yangzhou, Jiangsu, China; ^2^ Research Department for Agro-ecological Security, Jiangsu Coastal Area Institute of Agricultural Sciences, Yancheng, Jiangsu, China; ^3^ Faculty of Horticultural Science and Technology, Suzhou Polytechnic Institute of Agriculture, Suzhou, Jiangsu, China; ^4^ College of Environmental Science and Engineering, Yangzhou University, Yangzhou, Jiangsu, China; ^5^ State Key Laboratory of Soil and Sustainable Agriculture, Institute of Soil Science, Chinese Academy of Sciences, Nanjing, Jiangsu, China

**Keywords:** free-air CO_2_ enrichment, rice, source and sink, mineral elements, absorption and distribution

## Abstract

**Introduction:**

This study explores how elevated CO_2_ concentration may alter the source-sink dynamics in rice by providing additional carbon for photosynthesis, thereby affecting nutrient absorption and distribution.

**Methods:**

A free-air CO_2_ enrichment experiment was conducted on a japonica cultivar Wuyunjing 27 in 2017 and 2018 growing seasons. The plants were exposed to ambient and elevated CO_2_ level (increased by 200 μmol·mol-1) and two source-sink manipulation treatments (control with no leaf cutting and cutting off the top three leaves at heading).

**Results:**

The elevated CO_2_ significantly increased the above-ground biomass and the straw non-structural carbohydrate concentration by an average of 19.3% and 12.5%, respectively. Significant changes in the concentrations of N, S, Fe, and Zn in straw were noted under elevated CO_2_, with average decreases by 7.1, 7.2, 11.6, and 10.1%, respectively. The exposure to elevated CO_2_ significantly enhanced the elements accumulation, yet it had minimal impact on their distribution across different organs. When compared to intact rice, removing the top three leaves at heading reduced the above-ground biomass by 36.8% and the straw non-structural carbohydrate content by 44.8%. Leaf-cutting generally increased the concentration of elements in stem, leaf, and grain, likely due to a concentration effect from reduced biomass and carbohydrate accumulation. Leaf-cutting reduced element accumulation and shifted element allocation in rice organs. It increased the proportion of elements in stems while reduced their presence in leaves and grains.

**Discussion:**

Our study suggests that a dilution effect may cause a decrease in mineral elements concentrations under elevated CO_2_ because of the increase in biomass and carbohydrates.

## Introduction

Atmospheric carbon dioxide (CO_2_) levels have risen significantly from approximately 280 μmol·mol^-1^ during the Industrial Revolution to about 420 μmol·mol^-1^ today (NOAA, 2024), with projections suggesting a rise to 550 μmol·mol^-1^ by 2050 (IPCC, 2021). As a critical substrate for photosynthesis, elevated CO_2_ (eCO_2_) has profound implications for rice, one of the main stable food crops globally (Fan et al., 2016). Although eCO_2_ can enhance rice yield (Hu et al., 2021, Hu et al., 2022a; Ainsworth and Long, 2021), it often reduces the concentration of crucial mineral elements such as N, S, Mg, Fe, Cu, and Zn (Hu et al., 2022b; Hu et al., 2024). This reduction could pose ‘invisible hunger’ risks (Loladze, 2014) and impact the biochemical interactions between rice and soil, thus influencing overall soil health and sustainability. Given these factors, investigating the dynamics of mineral element absorption and distribution in rice under eCO_2_ conditions is vital for ensuring food security and improving the quality of arable land in the face of climate change.

Leaves under eCO_2_ conditions produce increased non-structural carbohydrates (NSC) (Zhu et al., 2016), which are transferred to the grains during the filling stage, resulting in a ‘dilution effect’ that associated with the reduction of concentrations of elements such as N, Fe, S and Zn (Zhang et al., 2013; Myers et al., 2014; Chaturvedi et al., 2017; Zhu et al., 2018; Ziska, 2022; Gojon et al., 2023). However, findings from free air CO_2_ enrichment (FACE) experiments have shown mixed outcomes. Some studies found no significant decrease in mineral element concentration in grains, while others reported increased concentrations of certain elements (Lieffering et al, 2004; Wang et al., 2014). This variability highlights the need for ongoing research in this domain, especially considering the potential influence of climatic variations across different years (Yang et al., 2007; Li et al., 2023).

Several studies conducted under FACE conditions have shown a general trend of increased accumulation of elements such as Fe, Mn, Ca, Mg, and Zn in rice stems and panicles but not in leaves (Lieffering et al, 2004; Zhou et al., 2021; Cui et al., 2023; Wang et al., 2023). Furthermore, studies suggest an enhanced distribution of these elements to rice panicle, while their presence in stem and leaf diminishes (Li et al., 2020; Jiang et al., 2020a). A study found that potassium (K) distribution in leaf decreased significantly by 16.3% under eCO_2_ conditions, while no substantial change was observed in stem and panicle (Pang et al., 2005). This indicates differential responses among elements to eCO_2_. The stem and leaf, which exposed to high CO_2_ concentrations more directly and longer than grain enclosed in a glume shell, may be more affected by eCO_2_. According to a study by Ujiie et al (2019), the concentrations of S, Mg, and Mn in straw decreased by 1.5-4 times as much as in grain under eCO_2_ conditions. Nutrient elements in grains are absorbed from the soil by roots and initially accumulate in vegetative organs such as stems and leaves before being transferred to grain. Therefore, to understand the changes in grain element composition by eCO_2_, it is essential to study the element accumulation and distribution in vegetative organs in the response to high CO_2_ concentrations. However, research in this area is limited and has mainly focused on nitrogen (Li et al., 2015), with less attention given to other elements.

The increase in atmospheric CO_2_ concentration has significant implications for rice photosynthesis (Dingkuhn et al., 2020; Fabre et al., 2020; Yin et al., 2022) and the absorption and transport of elements within the plant (Wei et al., 2018; Guo et al., 2015). Prior FACE studies showed that the concentrations of elements (such as N and Cu) in grain decreased under eCO_2_ while increased under partial defoliation treatment (Gao et al., 2021a), demonstrating the influence of alterations in the source-sink relationship on grain element concentrations. The accumulation of nutrients in grain during the grain-filling stage is mainly accomplished through mineral remobilization from vegetative organs and/or uptake by roots (Wu et al., 2010; Yuan et al., 2012). According to Sperotto et al. (2013), reducing source-sink ratio by flag leaf removal at anthesis does not change Fe and Zn concentration or content in mature seeds. It is possible that flag leaves are preferential but are not essential as a source of metals, which can probably be compensated by other leaves remobilization and/or continuous uptake by roots. However, it is not clear whether this assumption remains viable in the case of a significant increase in carbohydrate release. Moreover, it remains unclear how the source-sink relationship influences element absorption and distribution throughout the rice plant under eCO_2_ concentrations. This study employed a FACE setting with two distinct CO_2_ levels, ambient CO_2_ (aCO_2_) and elevated CO_2_ (eCO_2_, aCO_2_ + 200 μmol·mol^-1^), and established two source-sink ratio treatments: a conventional source-sink ratio (CK) and a reduced source-sink ratio by cutting off the top three leaves (LC). The investigation, conducted over two consecutive years, aims to elucidate the impacts of elevated CO_2_ and source-sink relationships on mineral elements uptake and distribution in rice.

## Materials and methods

### Experimental site and FACE system

**Figure 1:**
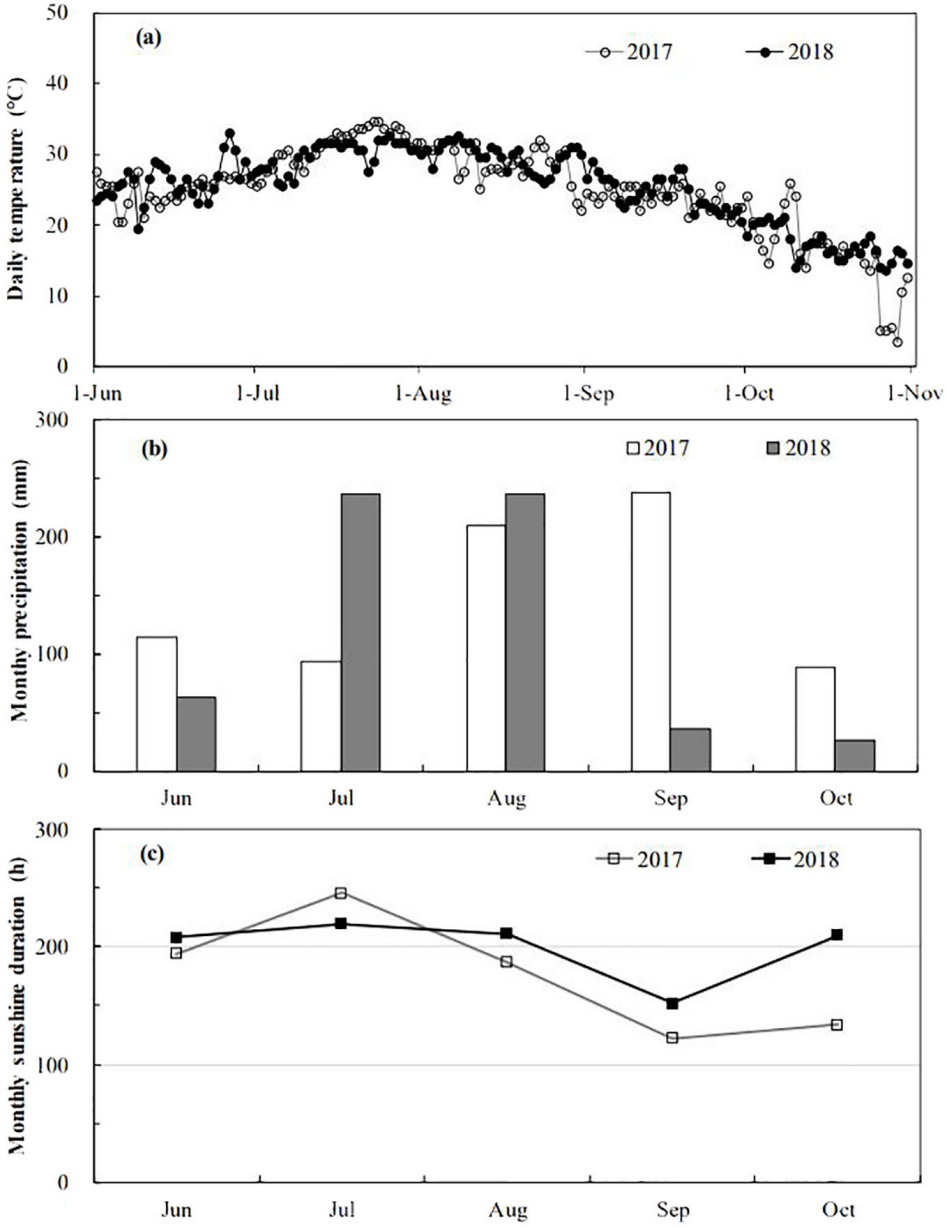
Daily mean temperature **(A)**, monthly mean precipitation **(B)** and monthly mean sunshine duration **(C)** at the experiment site during 2017-2018 growing seasons.

This study was conducted at the FACE system located in Yangzhou (119°42′0″E, 32°35′5″N), Jiangsu Province, China. The site featured a soil composition of organic carbon (24.8 g·kg^-1^), total nitrogen (1.13 g·kg^-1^), total phosphorus (0.54 g·kg^-1^), and total potassium (9.7 g·kg^-1^), with available nitrogen, phosphorous, and potassium concentrations of 122.4 mg·kg^-1^, 15.1 mg·kg^-1^, and 56.5 mg·kg^-1^ respectively, and a pH of 6.9. The FACE system comprised six plots distributed across paddies with similar soil characteristics and agronomic history. Three plots were randomly assigned to elevated CO_2_ treatment and three to ambient conditions, each measuring approximately 80 m^2^. To prevent CO_2_ cross-contamination, the centers of the FACE and ambient plots were spaced 90 meters apart. Pure CO_2_ was delivered into the center of the FACE plots through perimeter pipelines. CO_2_ concentrations were controlled by a computer system adjusting for atmospheric levels, wind direction, and speed, ensuring a consistent 200 μmol·mol^-1^ above ambient CO_2_ levels at crop canopy height throughout the main growth period of rice. CO_2_ fumigation was conducted daily from seedling transplantation to maturity, from sunrise to sunset. The average daily temperatures from June 1 to October 31 were 24.9°C in 2017 and 25.5 °C in 2018. The average monthly precipitation was 149.1 mm in 2017 and 120.1 mm in 2018, with monthly sunshine durations of 176.5 and 200.2 hours, respectively, as detailed in **Figure 1**.

### Crop cultivation and defoliation treatment

In each experimental season, the *japonica* cultivar Wuyunjing 27, a popular cultivar in the study region, was used. Seeds were sown in a nursing paddy, and seedlings were grown for one month under ambient conditions. Seedlings were manually transplanted to the experimental plots at a density of two per hill around June 20 each season. Fertilization included urea (46% N) and a balanced NPK compound (N: P_2_O_5_: K_2_O = 15:15:15) applied at a rate of 22.5 g N·m^-2^. In each growing season, nitrogen was administered into three applications: 40% as a basal dressing one day before transplanting, 30% as a top dressing at the early tillering stage, and 30% as a top dressing at panicle ignition. Phosphorus and potassium were also applied as compound fertilizers P_2_O_5_ and K_2_O at a rate of 9 g·m^-2^ one day before transplanting. The water regime was meticulously managed with paddy fields submerged to a depth of 5 cm from about June 17 to July 20, followed by a period of wet-dry cycles from about July 21 to August 10 through natural drainage and intermittent irrigation. Routine pest and disease management was routinely conducted throughout the growing seasons. Fertilizer application and water regime specifics are available in Gao et al. (2021a).

At the heading stage, 30 plants per plot were selected for the study. To investigate source-sink dynamics, half of these plants underwent a leaf-cutting treatment there the top three leaves were removed (LC), and the other half were left intact as controls (CK). This method ensured uniformity in tiller number and developmental stage across treatments, critical for evaluating the impact of altered source-sink relationships on growth and yield.

### Plant sampling and parameter measurements

At maturity, five hills, each representing the average number of tillers per hill, were selected for destructive sampling. These samples were systematically divided into leaves, stems, and panicles. To determine the accurate dry weights, stems and leaves were oven-dried at 105 °C for 30 min, followed by 80 °C for 72 h. Panicles were air-dried to a constant weight. After drying, panicles were carefully threshed and dehulled to produce brown rice. Above-ground biomass was calculated from the dry weights of each organ. Stems and leaves were ground to less than 0.15 mm using a stainless-steel mill, and brown rice was ground into powder using a vibration disk mill (TS1000, Germany) with a 100-mesh sieve, preparing them for subsequent analyses.

The non-structural carbohydrate (NSC) content was determined as the sum of total soluble sugars and starch. The anthrone H_2_SO_4_ method, as described by Yoshida et al. (1976), was employed to measure soluble sugars and starch in the stems and leaves. Nitrogen (N) concentrations were determined using a Kjeltec 8400 autoanalyzer (FOSS Analytical AB, Sweden) after hydrolysis by the Kjeldahl method. The concentrations of other elements were determined as follows: 0.50 g of flour was weighed into the lining tube of a microwave digestion apparatus (MARS5, CEM Corporation, Matthews, USA), followed by the addition of 5 mL of 65% nitric acid, 3 mL of ultrapure water, and three drops of hydrogen peroxide; the mixture was then placed into a microwave digestion-meter for high-temperature digestion. Post-digestion, the volume of the digestion liquid was increased to 50 mL and filtered using quantitative filter paper. The filtrate’s mineral content was analyzed using an inductively coupled plasma emission spectrometer (iCAP6300, Thermo Fisher Scientific, USA). This analysis included the macroelements phosphorus (P), sulfur (S), potassium (K), calcium (Ca), and magnesium (Mg) and the microelements boron (B), copper (Cu), iron (Fe), manganese (Mn), and zinc (Zn). Element accumulations were calculated by multiplying the biomass of each organ by its elemental concentration. Elemental allocations were then determined based on the total accumulation across all sampled organs.

### Statistical analyses

The field experiment was designed as a completely randomized setup with a split-plot arrangement. The CO_2_ levels were treated as the main plot variable, while source-sink treatments acted as subplots, each with three replications. Analysis of variance (ANOVA) was conducted using the SPSS statistical software (Version 20.0, SPSS Inc., Chicago, USA) to assess the effects of these treatments. *Post-hoc* comparisons among treatment means were performed using the Least Significant Difference (LSD) test. Statistical significance of the effects was indicated as follows: highly significant (** *P* < 0.01), significant (* *P* < 0.05), and marginally significant (+ *P* < 0.1). Additionally, Pearson’s correlation coefficients were calculated to explore the relationships among the different measured parameters.

## Results

### Above-ground biomass response

**Figure 2:**
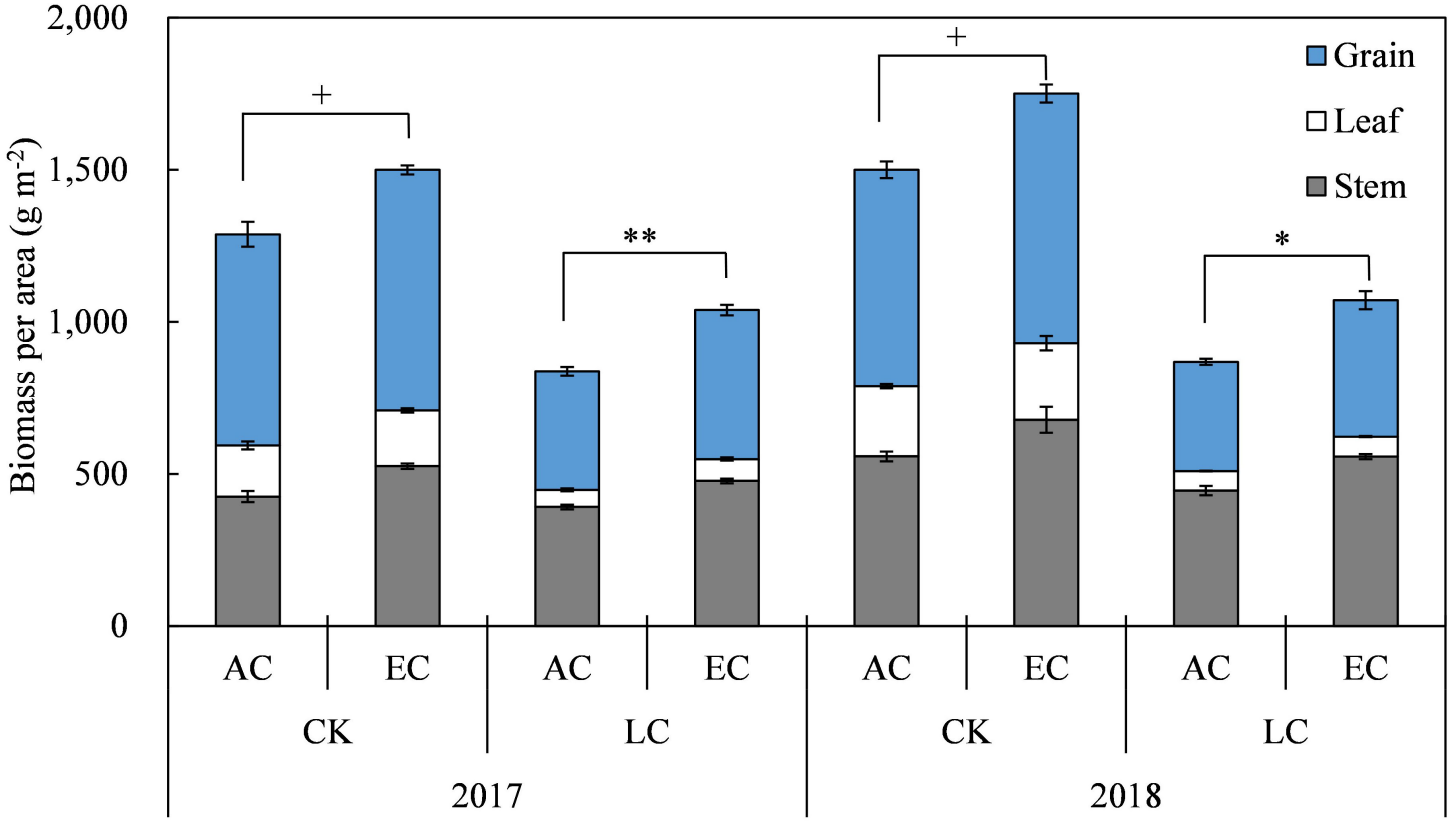
Response of rice biomass at maturity to elevated CO_2_ under CK (no leaf cutting) and LC (cutting off top three leaves at heading) in 2017 and 2018 growing seasons. Each bar in the figure represents the mean values across three plots for ambient CO_2_ (AC) or elevated CO_2_ (EC, AC + 200 μmol·mol^-1^); vertical bars represent standard error (n = 3). Statistically significant effects are indicated as ** *P* < 0.01; * *P* < 0.05; + *P* < 0.1.

In the present two-year study, the above-ground biomass (AGB) of rice was higher in 2018 than in 2017 (**Figure 2** and **Table 1**). As compared to the ambient, on average, eCO_2_ increased the AGB by19.3% significantly, and the increase in 2017 and 2018 were close to each other. Among the different plant organs, eCO_2_ increased the biomass of stem, leaf, and grain by 23.0%, 10.3%, and 18.3%, respectively. The magnitude of AGB increase by eCO_2_ was larger for LC crops (+23.7%) compared to CK crops (+16.6%), though no interaction of CO_2_ with LC was detected (**Table 1**). Compared with CK crops, the LC treatment significantly reduced AGB by 36.8% on average, with reductions of 32.7% and 40.3% observed in 2017 and 2018, respectively (**Table 1**).

**Table 1:** Analysis of variance (ANOVA) results of effects of elevated CO_2_ and LC (leaf-cutting treatment at heading) on rice biomass at maturity in 2017 and 2018 growing seasons.

ANOVA	Stem	Leaf	Grain	Aboveground biomass
CO_2_	**<0.001**↑	0.088↑	**<0.001**↑	**<0.001**↑
LC	**<0.001**↓	**<0.001**↓	**<0.001**↓	**<0.001**↓
Year	**<0.001**↑	**<0.001**↑	0.736	**<0.001**↑
CO_2_ × LC	0.673	0.562	0.815	0.663
CO_2_ × Year	0.406	0.784	0.974	0.759
LC × Year	**0.013**	**<0.001**	0.113	**0.007**
CO_2_ × LC × Year	0.912	0.505	0.747	0.778

↑ and ↓ represent the positive and negative effects of CO_2_ or leaf-cutting, respectively, or represent increase and decrease in 2018 compared to 2017, respectively.

The bold was used to highlight values less than 0.1.

### Element concentrations

**Figure 3:**
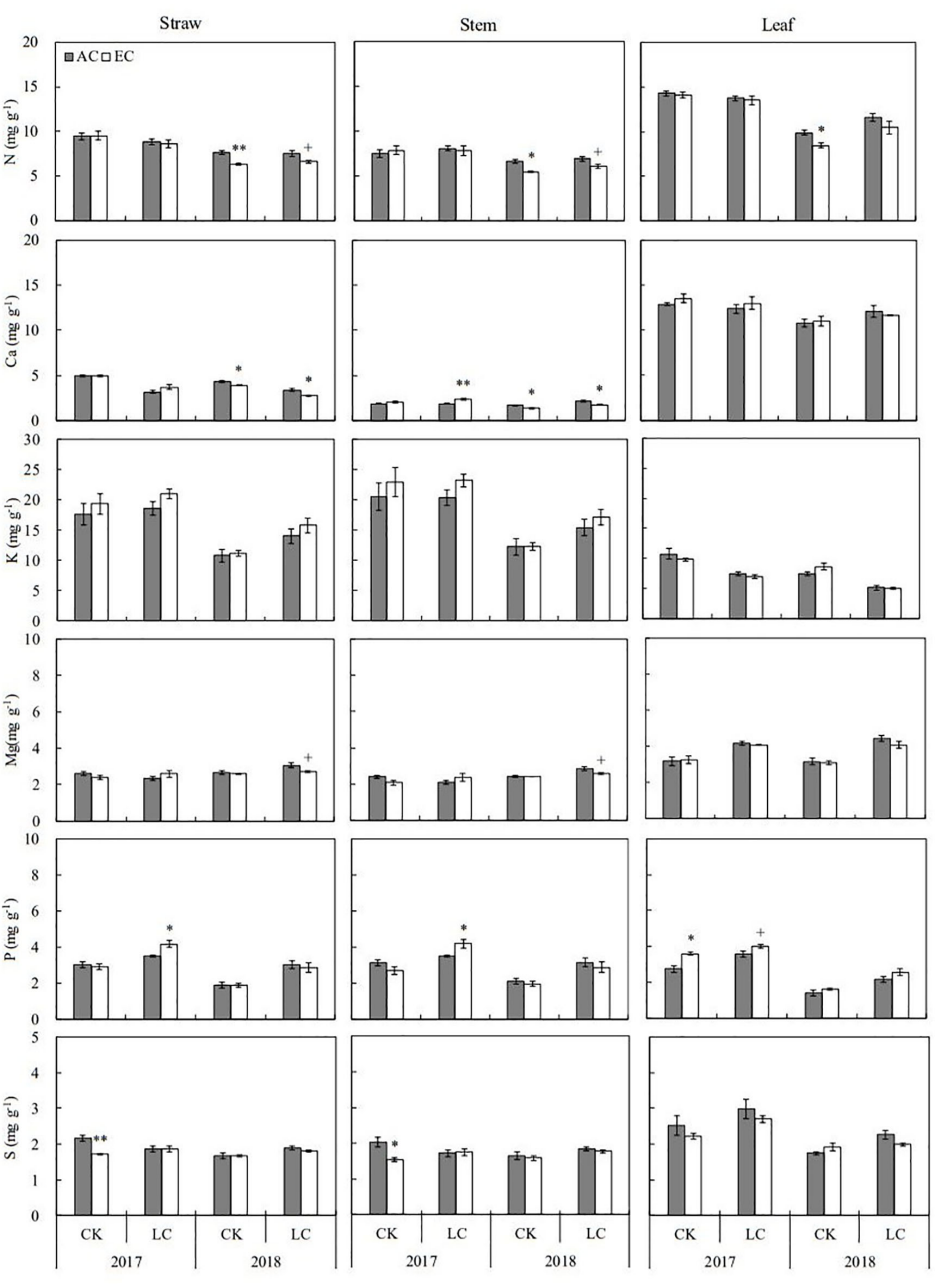
Response of macroelements concentrations in rice straw, stem, and leaf at maturity to elevated CO_2_ under CK (no leaf cutting) and LC (cutting off top three leaves) in 2017 and 2018 growing seasons. Each bar in the figure represents the mean values across three plots for ambient CO_2_ (AC) or elevated CO_2_ (EC, AC + 200 μmol·mol^-1^); vertical bars represent standard error (n = 3). Statistically significant effects are indicated as ** *P* < 0.01; * *P* < 0.05; + *P* < 0.1.

**Figure 4:**
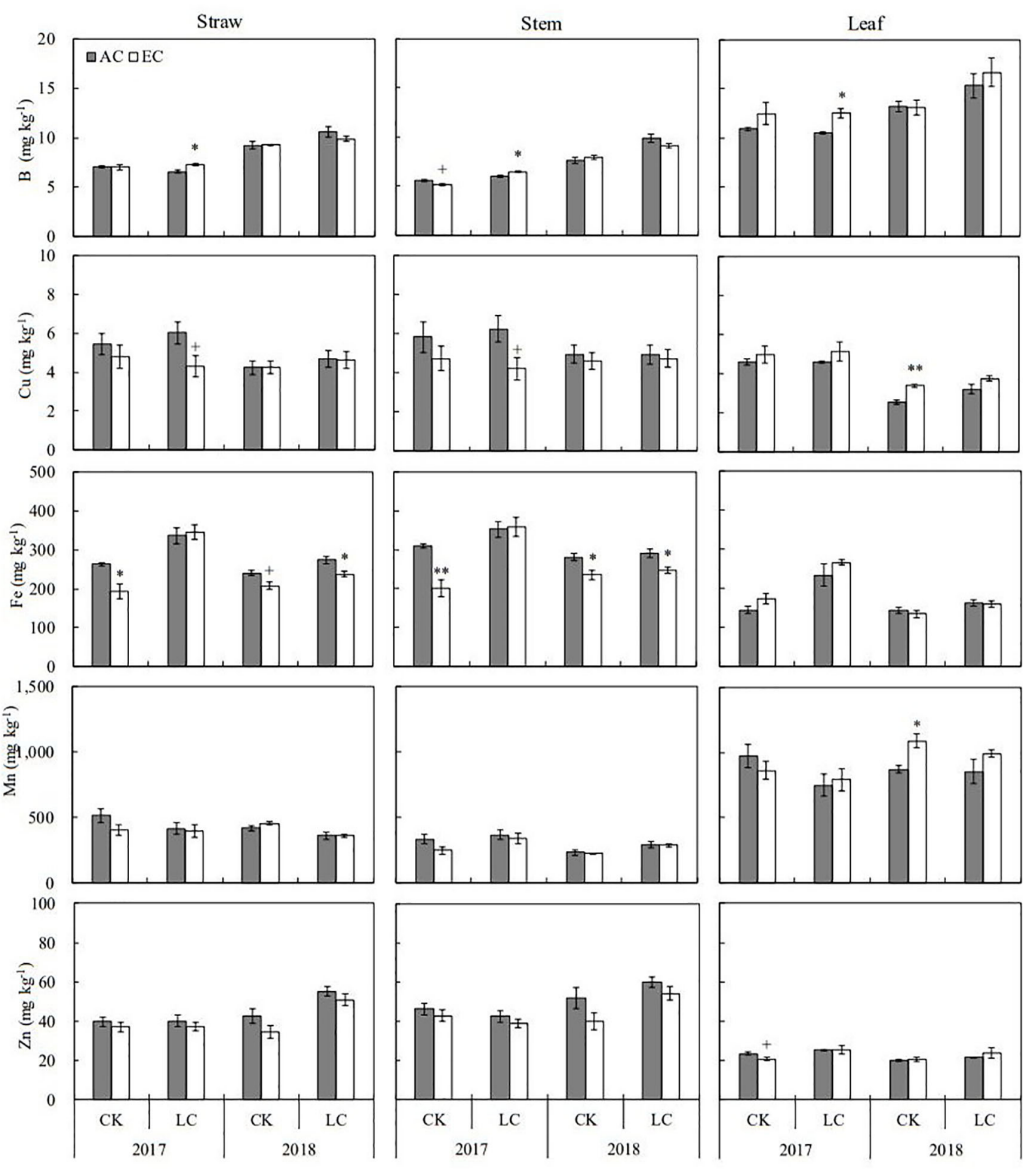
Response of microelements concentrations in rice straw, stem, and leaf at maturity to elevated CO_2_ under CK (no leaf cutting) and LC (cutting off top three leaves) in 2017 and 2018 growing seasons. Each bar in the figure represents the mean values across three plots for ambient CO_2_ (AC) or elevated CO_2_ (EC, AC+200 μmol·mol^-1^); vertical bars represent standard error (n = 3). Statistically significant effects are indicated as ** *P* < 0.01; * *P* < 0.05; + *P* < 0.1.

The concentrations of eleven mineral elements including N, Ca, K, Mg, P, S, B, Cu, Fe, Mn, and Zn in rice straw and grain were determined in the present study (**Figures 3**, **4**; **Supplementary Tables S1**, **S2**). Apart from Mg, B, and Zn, the concentrations of other elements in straw were lower in 2018 than in 2017. Relative to the concentrations of grain elements in 2017, higher K, Mg, P, and B concentrations but lower N, Ca Fe, Mn, and Zn concentration were found in 2018. Among these elements, N, S, Fe, and Zn concentrations in straw were significantly altered by eCO_2_ when averaged across different source-sink crops in two years, with average decreases by 7.1, 7.2, 11.6, and 10.1%, respectively. Response of these elements in grain was not significant over the growing seasons of 2017 and 2018 (**Supplementary Table S1**). Compared to CK crops, the concentrations of N, K, Mg, P, S, and Zn in grain showed a significant increase in response to LC treatment, ranging from 3.6% to 15.1% on average, similar responses of stem and leaf were observed in two seasons.

### Non-structural carbohydrates

**Figure 5:**
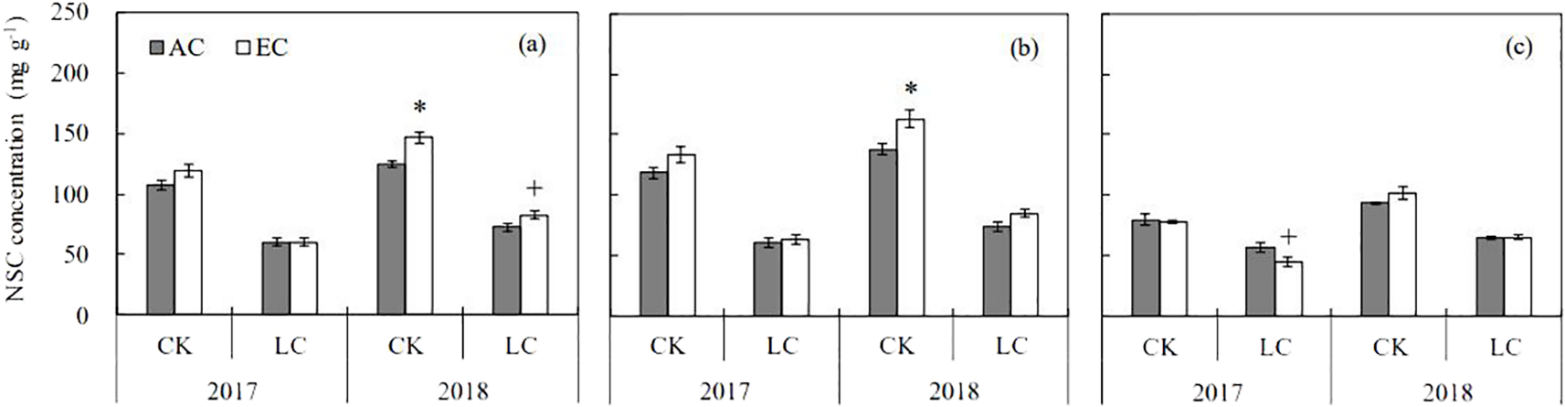
Response of non-structural carbohydrate (NSC) concentrations in rice straw **(A)**, stem **(B)**, and leaf **(C)** at maturity to elevated CO_2_ under CK (no leaf cutting) and LC (cutting off top three leaves) in 2017 and 2018 growing seasons. Each bar in the figure represents the mean values across three plots for ambient CO_2_ (AC) or elevated CO_2_ (EC, AC + 200 μmol·mol^-1^); vertical bars represent standard error (n = 3). Statistically significant effects are indicated as * *P* < 0.05; + *P* < 0.1.

On average, eCO_2_ increased straw non-structural carbohydrates (NSC) content by 12.5% (**Figure 5**), with a more pronounced CO_2_ effect occurring in 2018 (+16.2%, *P* < 0.01) than in 2017 (+7.3%, *P* > 0.1), which resulted in a significant CO_2_ × year interaction (**Supplementary Table S2**). In addition, this parameter showed a significant CO_2_ × organ interaction: it was increased in stem by 14.2% (*P* < 0.01) by eCO_2_, but had no effects in leaf. A strong LC effect was observed on straw NSC content, with a large decrease by 46.8% and 42.7% in 2017 and 2018, respectively. Under the LC treatment, the NSC decrease was greater in stem (-49.0%) than in leaf (-35.7%), with a notable significant LC × organ interaction (**Supplementary Table S2**).

### Correlation analysis

**Figure 6:**
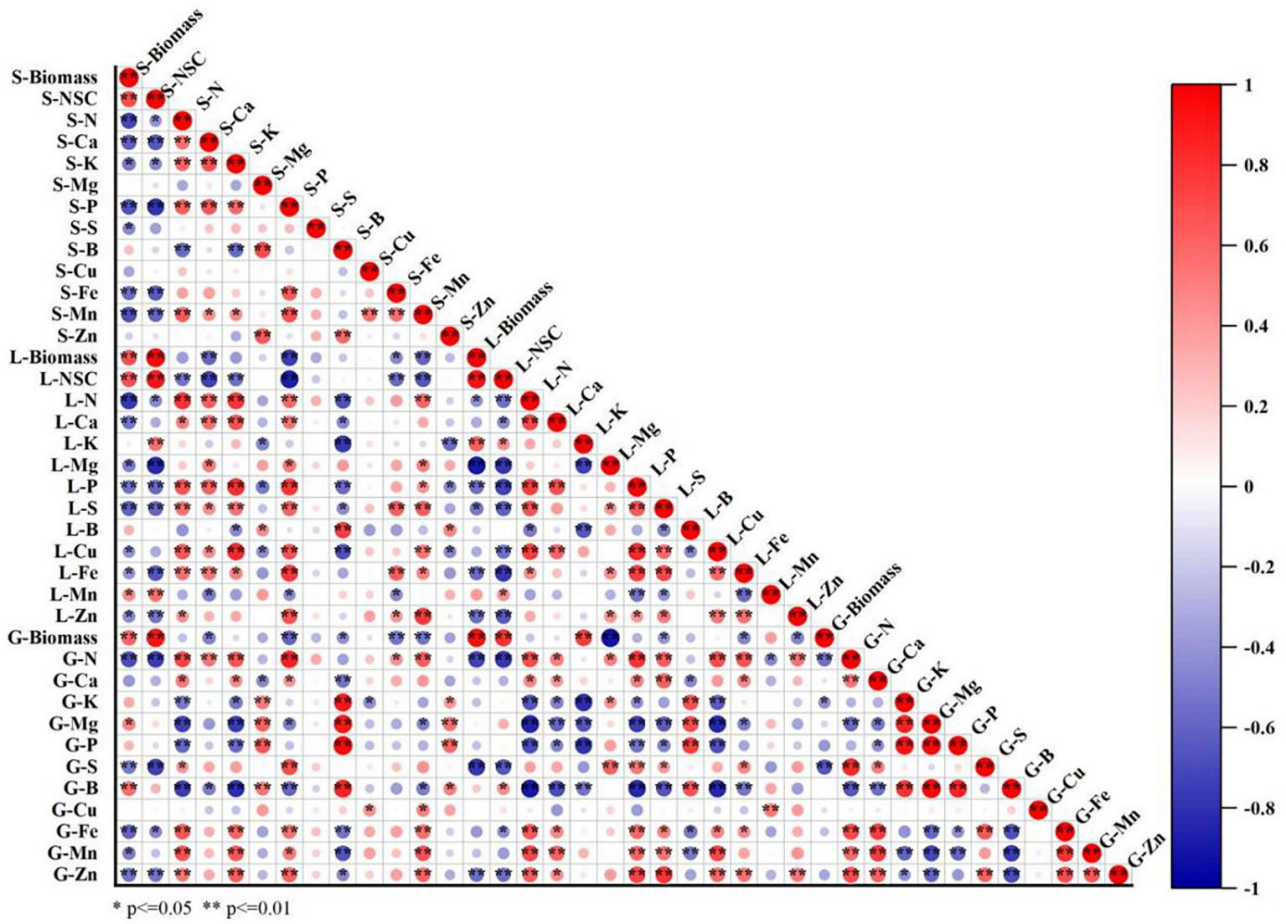
Correlation analysis of elements concentration in different parts of rice with biomass, non-structure carbohydrate (NSC) concentrations of stem and leaf under CO_2_ and leaf-cutting treatments. Red and blue showed significant positive and negative correlation, while white showed no significant correlation. S-Biomass, L-Biomass, and G-Biomass represent stem, leaf, and grain biomass respectively. S-NSC and L-NSC represent NSC concentrations in stem and leaves, respectively. S-N, S-Ca, S-K, S-Mg, S-P, S-S, S-B, S-Cu, S-Fe, S-Mn, and S-Zn represent concentrations of N, Ca, K, Mg, P, S, B, Cu, Fe, Mn, and Zn in stem, respectively. L-N, L-Ca, L-K,L-Mg, L-P, L-S, L-B, L-Cu, L-Fe, L-Mn, and L-Zn represent concentrations of N, Ca, K, Mg, P, S, B, Cu, Fe, Mn, and Zn in leaves, respectively. G-N, G-Ca, G-K, G-Mg, G-P, G-S, G-B, G-Cu, G-Fe, G-Mn, and G-Zn represent concentrations of N, Ca, K, Mg, P, S, B, Cu, Fe, Mn, and Zn in grain, respectively. * and ** indicate significance at *P* < 0.05, and *P* < 0.01, respectively. (n = 24).

Pearson correlation analyses were conducted to investigate the relationship between the concentrations of elements and the biomass or NSC in different organs (**Figure 6**). The concentrations of N, Ca, K, P, S, Fe, and Mn in stems and leaves were negatively correlated with biomass and NSC, whereas concentrations of N, S, Fe, Mn, and Zn in grain were negatively correlated with grain yield. In contrast, a significant positive linear correlation between biomass and NSC concentration was observed in stems and leaves.

### Element accumulation and allocation

We multiplied the elemental concentration by biomass of each organ to calculate the total amounts of the various elements removed by harvesting (**Table 2**). Compared with the ambient, eCO_2_ significantly increased accumulation of elements in both years, with the increase ranging from 5.8% to 29.9% on average. In contrast, the LC treatment significantly decreased the accumulation of mineral elements, with similar magnitude in 2017 and 2018.

**Table 2:** Response of mineral elements accumulations of rice aboveground to elevated CO_2_ and LC (cutting off top three leaves at heading) in 2017 and 2018 growing seasons.

Year	Treatment	CO_2_	N (g m^-2^)	Ca (g m^-2^)	K (g m^-2^)	Mg (g m^-2^)	P (g m^-2^)	S (g m^-2^)	B (mg m^-2^)	Cu (mg m^-2^)	Fe (mg m^-2^)	Mn (mg m^-2^)	Zn (mg m^-2^)
2017	CK	AC	16.6 ± 0.7	3.1 ± 0.2	12.6 ± 1.7	2.5 ± 0.1	4.6 ± 0.3	2.2 ± 0.2	5.3 ± 0.4	6.8 ± 0.6	181.6 ± 10.3	335.0 ± 23.0	44.7 ± 2.8
		EC	19.3 ± 1.0	3.7 ± 0.1	16.1 ± 1.1	2.8 ± 0.1	5.2 ± 0.2	2.2 ± 0.1	6.5 ± 0.4	6.7 ± 0.9	165.9 ± 17.1	322.6 ± 35.1	49.4 ± 2.4
		% Change	16.2 +	19.6 *	27.9 ns	10.3 ns	14.4 +	2.9 ns	21.5 ns	-1.8 ns	-8.6 ns	-3.7 ns	10.6 ns
	LC	AC	10.9 ± 0.6	1.5 ± 0.1	9.6 ± 0.4	1.6 ± 0.1	3.2 ± 0.1	1.4 ± 0.1	3.7 ± 0.1	4.7 ± 0.5	168.4 ± 8.4	202.7 ± 18.6	31.3 ± 1.7
		EC	13.2 ± 0.8	2.2 ± 0.1	13.2 ± 0.4	2.1 ± 0.1	4.4 ± 0.2	1.7 ± 0.1	4.8 ± 0.1	4.4 ± 0.5	209.1 ± 10.9	241.1 ± 24.8	35.7 ± 1.8
		% Change	20.6 +	43.7 *	38.2 **	31.4 **	36.4 **	22.6 *	29.1 **	-6.4 ns	24.2 *	18.9 ns	14.0 ns
2018	CK	AC	15.2 ± 0.4	3.6 ± 0.1	11.0 ± 1.0	3.4 ± 0.2	4.8 ± 0.2	2.2 ± 0.1	11.2 ± 0.3	6.80 ± 0.9	211.0 ± 5.1	354.9 ± 22.0	49.5 ± 3.3
		EC	16.5 ± 0.9	3.8 ± 0.3	13.3 ± 0.9	3.9 ± 0.2	5.6 ± 0.3	2.5 ± 0.1	13.1 ± 0.6	8.07 ± 0.8	216.1 ± 23.2	453.8 ± 33.0	53.3 ± 1.9
		% Change	8.7 ns	7.4 ns	20.4 ns	14.7 +	16.0 +	17.1 +	17.0 *	18.7 ns	2.4 ns	27.9 +	7.7 ns
	LC	AC	9.5 ± 0.31	1.8 ± 0.1	8.4 ± 0.5	2.3 ± 0.1	3.4 ± 0.1	1.4 ± 0.1	7.2 ± 0.2	4.2 ± 0.3	150.4 ± 9.6	196.6 ± 19.1	37.8 ± 2.3
		EC	11.0 ± 0.63	1.8 ± 0.1	11.5 ± 0.9	2.6 ± 0.1	4.1 ± 0.3	1.7 ± 0.1	8.6 ± 0.1	5.1 ± 0.4	161.3 ± 7.3	242.0 ± 10.2	44.6 ± 3.2
		% Change	15.2 ns	1.5 ns	36.1 *	14.0 +	21.3 +	18.5 +	18.8 **	22.4 ns	7.3 ns	23.1 ns	18.1 ns
ANOVA results (*P* value)													
CO_2_			**0.001**↑	**< 0.001**↑	**< 0.001**↑	**< 0.001**↑	**< 0.001**↑	**0.001**↑	**< 0.001**↑	0.332	0.270	**0.025**↑	**0.013**↑
LC			**< 0.001**↓	**< 0.001**↓	**0.001**↓	**< 0.001**↓	**< 0.001**↓	**< 0.001**↓	**< 0.001**↓	**< 0.001**↓	**0.031**↓	**< 0.001**↓	**< 0.001**↓
Year			**< 0.001**↓	0.158	**0.015**↓	**< 0.001**↑	0.459	**0.088**↑	**< 0.001**↑	0.378	0.706	**0.051**↑	**0.003**↑
CO_2_ × LC			0.876	0.629	0.731	0.847	0.446	0.588	0.511	0.776	0.103	0.971	0.707
CO_2_ × Year			0.284	**0.019**	0.487	0.881	0.571	0.308	0.255	0.160	0.802	0.105	0.832
LC × Year			0.721	**0.100**	0.555	**0.007**	0.278	0.358	**< 0.001**	0.515	**< 0.001**	**0.038**	0.367
CO_2_ × LC × Year			0.774	0.447	0.805	0.170	0.361	0.182	0.598	0.933	0.179	0.150	0.638

AC and EC refer to ambient CO_2_ and elevated CO_2_, respectively. CK and LC refer to no leaf cutting and cutting off top three leaves, respectively. Values are means ± standard error (n = 3). Statistically significant effects are indicated as + *P* < 0.1;** *P* < 0.01;* *P* < 0.05; ns, not significant. ↑ and ↓ represent the positive and negative effects of CO_2_ or LC, respectively, or represent increase and decrease in 2018 compared to 2017, respectively.

The bold was used to highlight values less than 0.1.

**Figure 7:**
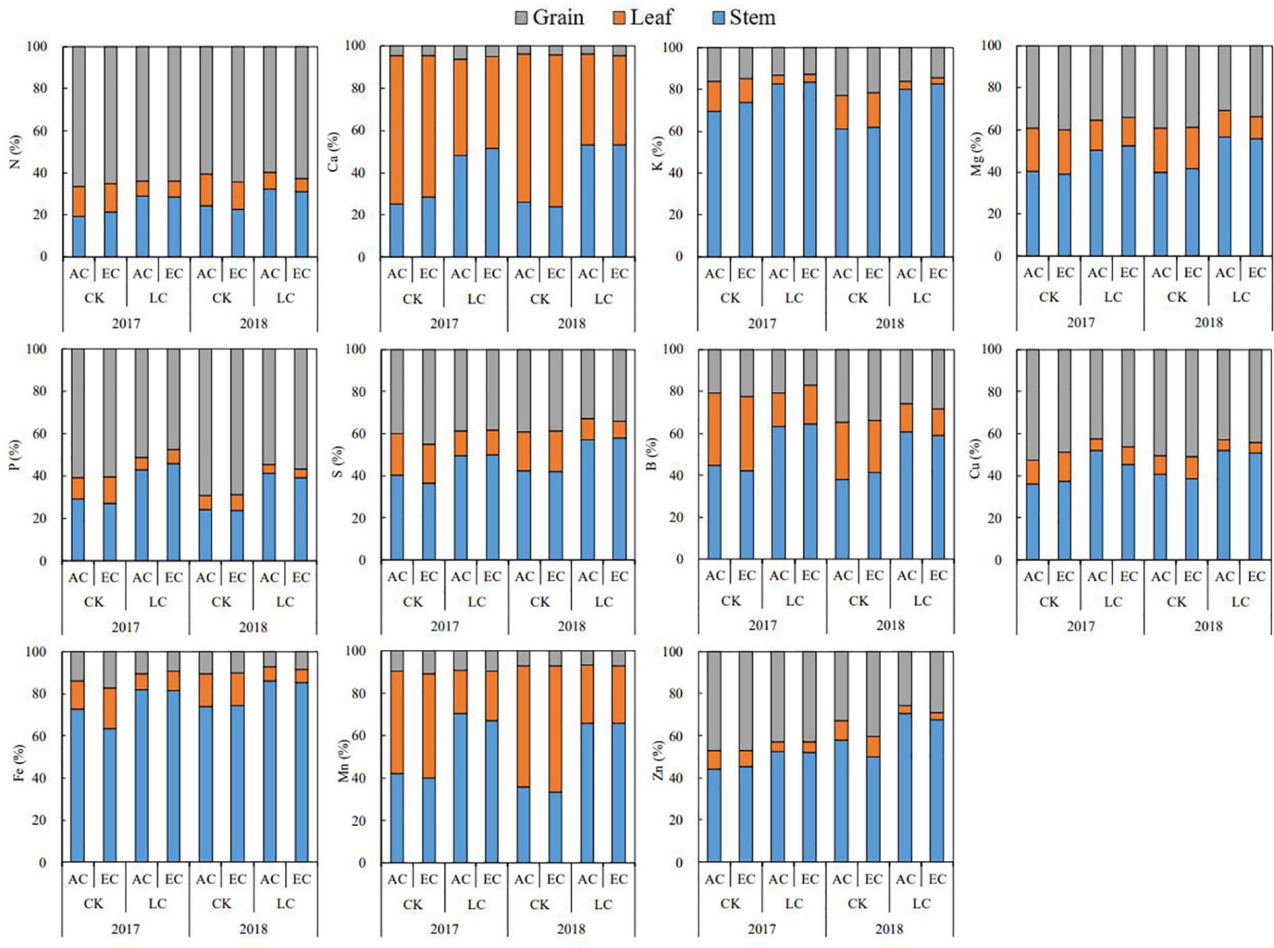
The mineral elements allocations in rice stem, leaf, and grain as affected by elevated CO_2_ and LC (cutting off top three leaves) in 2017 and 2018 growing seasons. AC and EC refer to ambient CO_2_ and elevated CO_2_, respectively. CK and LC refer to no leaf cutting and cutting off top three leaves, respectively. Each bar in the figure represents the mean values across three plots for ambient CO_2_ or elevated CO_2_.

The effects of all main factors (CO_2_, year, and LC) on the elements distributions at maturity differed among the three organs (**Figure 7** and **Supplementary Table S3**). Averaged across two years and different source-sink ratio crops, eCO_2_ significantly increased Zn allocation to grain by 7.2%, and Cu and Fe allocation to leaf by 22.1% and 16.1%, while respectively decreased Fe and Mn allocations to stem by 3.1% and 3.7%. Significant year effects were observed on the elements percentages of each organs at maturity (**Supplementary Table S3**). Relative to these elements fractions in 2017, higher fractions of grain K, P, and B, and stem K, Mn, and Zn, and leaf K and Mn were found in 2018. As compared to CK crops, LC treatment significantly altered the elements distributions. Overall, LC treatment significant increased elements allocation to stem but decreased those to grain and leaf, and similar trends were seen in 2017 and 2018, though interactions of LC with year were detected on some elements allocations (**Supplementary Table S3**).

## Discussion

### Above-ground biomass

Increasing atmospheric CO_2_ concentration has a fertilization effect that contributes to rice growth, as CO_2_ is essential for photosynthesis. Numerous studies consistently demonstrate this effect, including those in artificial climate chambers, open-top chambers (OTCs), and free air CO_2_ enrichment (FACE) platforms (Ainsworth and Long, 2021). Among these platforms, FACE is generally believed that FACE is closer to the natural ecological environment, thus truly reflecting the growth of rice under future elevated CO_2_ conditions. A meta-analysis of 20 years FACE studies suggested that, on average, elevated CO_2_ (eCO_2_) increased above-ground biomass of rice by 19%, and the increase is generally more pronounced in panicles than in stems or leaves (Hu et al., 2022a). From the perspective of grain yield alone, an average increase of 16% under eCO_2_ conditions was observed by Hu et al. (2021). The yield enhancement by elevated CO_2_ varied across different rice varieties (Ainsworth and Long, 2021; Yang et al., 2007; Kim et al., 2003), with hybrid rice (24.7%) was significantly greater than conventional rice (14.2%) (Hu et al., 2021). The leaf-cutting (LC) treatment led to a notable reduction in biomass across all above-ground plant parts due to decreased photosynthetic productivity and assimilate availability in the stem and panicle, culminating in reduced biomass.

While the ‘CO_2_ fertilization’ effect is well-documented to increase yields, the actual gains in field conditions were often lower than expected (Long et al., 2006; Ainsworth and Long, 2021; Sonnewald and Fernie, 2018). This discrepancy was increasingly attributed to the source-sink ratio of rice (Lv et al., 2020; Yin et al., 2022). Previous FACE studies have demonstrated that modifying the source-sink ratio by thinning flowers or cutting off leaves significantly affected rice yield response to eCO_2_ (Gao et al., 2021b). Specifically, reducing the source-sink ratio appeared more effective in enhancing biomass under eCO_2_, suggesting potential strategies for maximising yield benefits in future high CO_2_ conditions (Dingkuhn et al., 2020; Lawlor and Paul, 2014; Paul et al., 2020).

### Element concentration dynamics

Nitrogen (N) is an essential element of rice protein, an important element affecting leaf photosynthesis (Cai et al., 2020), and a nutrient element strongly influenced by elevated CO_2_ concentration (Gojon et al., 2023). Research has indicated that elevated CO_2_ enhanced the synergistic cycles of nitrogen and carbon (Cui et al., 2023), boosting the translocation of N from vegetative parts to grains (Xu et al., 2016; Jiang et al., 2020b), which consequently reduces its concentration in stems and leaves. Additionally, eCO_2_ often results in reduced stomatal conductance, diminishing leaf transpiration and potentially lowering N uptake due to decreased soil nutrient demand (Pang et al., 2006; Pozo et al., 2007). Besides N, in this study, we observed consistent decreases in S, Cu, Fe, Mn, and Zn under eCO_2_, which corroborates findings from other FACE studies (Uddling et al., 2018; Myers et al., 2014; Zhu et al., 2018). These declines are typically attributed to the ‘dilution effect’ (Pang et al., 2005; Chaturvedi et al., 2017; Loladze, 2014; Li et al., 2015; Ujiie et al., 2019; Jiang et al., 2020b), where increased plant biomass leads to lower nutrient concentrations. Correlation analysis confirmed significantly negative relationships between the biomass and the concentrations of N, S, Fe, and Mn in plant tissue.

Conversely, the concentrations of K and P increased, which does not align with the typical dilution effect narrative (Ren et al., 2007; Wang et al., 2023). K is crucial for carbohydrate synthesis and transport, and P, a key player in energy transfer and photosynthetic regulation (Ellsworth et al., 2022), has shown higher demand and uptake under eCO_2_ (Shimono et al., 2009; Wang et al., 2023). This suggests that eCO_2_ affects growth and modifies nutrient uptake priorities based on physiological needs, indicating a complex interaction between CO_2_ levels and nutrient dynamics that transcends simple biomass accumulation. Interestingly, the leaf-cutting (LC) treatment increased nearly all measured mineral element concentrations across different plant organs, contrasting with the broader biomass reduction and demonstrating a concentration effect due to reduced tissue mass. However, reductions in K and Mn in leaves under LC treatments suggest a decline in photosynthetic efficiency and sugar transport, which affects the demand for these specific nutrients.

The variability observed in element concentrations year-over-year also highlights the significant influence of annual climatic conditions on nutrient dynamics. For instance, the lower element content in stems in 2018 compared to 2017 could be linked to higher biomass production driven by less rainfall and more sunlight (Li et al., 2015; Wang et al., 2024), illustrating the intricate connections between environmental factors, plant physiology, and nutrient uptake under changing climatic conditions. This nuanced understanding of how elevated CO_2_ and defoliation affect nutrient concentrations provides valuable insights into managing crop nutrition in the face of global climate change, emphasizing the need for adaptive strategies that consider both environmental influences and intrinsic plant responses.

### Non-structural carbohydrate dynamics

Non-structural carbohydrates (NSCs) are crucial for plant growth, serving as primary metabolites that regulate energy and structural functions within plant systems. Consistent with Zhu et al. (2016) findings, an increase in NSC concentrations in rice straw was observed in the present study when exposed to eCO_2_, with increments ranging between 22-70%. Notably, our results indicated a lower increase in NSC concentration than previous studies, likely due to varietal differences. Specifically, the *japonica* rice exhibited a lesser response to eCO_2_ compared to the *indica* rice. From the perspective of source-sink balance, compared with CK crops, the NSC concentration of straw was significantly reduced by LC treatment. This variation could be attributed to the source-sink dynamics within the plants, where the top three functional leaves, crucial for photosynthetic activity, significantly influence NSC production and distribution. Under eCO_2_ conditions, enhanced CO_2_ levels act as a vital carbon source for photosynthesis, potentially leading to an increased synthesis of assimilates. However, if the sink capacity, primarily the grain, does not adequately accommodate this increased production, excess carbohydrates may accumulate in the straw, thereby limiting potential yield improvements.

### Elemental accumulation and allocation

Increased carbon capture under eCO_2_ conditions leads to greater carbohydrate accumulation in rice, while will the accumulation of mineral elements increase as a result? This study showed that elemental accumulation in rice enhanced with an increased carbon source provided by eCO_2_ yet diminishes when the source is decreased by LC. This trend aligns with biomass response patterns under both conditions. Notably, eCO_2_ has been shown to increase the absorption of N and P from the soil, altering the nutrient cycling and potentially impacting soil quality adversely (Cui et al., 2023; Zhang et al., 2022; Gojon et al., 2023). Over time, enhanced CO_2_ levels could significantly reduce soil available phosphorus, as demonstrated in a 15-year FACE study (Wang et al., 2023). The adventitious root length and adventitious root number could be increased under eCO_2_ by 25-37% (Yang et al., 2008), which influences the rhizosphere microbial environment and promotes soil organic matter decomposition, underscores rice dynamic changes in nutrient uptake (Drissner et al, 2007; Jin et al., 2014). These findings suggest that future strategies in rice cultivation should focus on improving soil organic matter and fertilizer utilization to mitigate nutrient elements loss under climate change conditions.

The distribution of mineral elements within rice plants is notably influenced by eCO_2_, with varying effects across different organs and varieties. This study indicates a shift in the allocation of elements like Fe, Mn, and Zn, with a reduction in stem and an increase in grain. These changes could be attributed to the interaction between CO_2_ levels and environmental factors across different years. Interestingly, while the proportion of elements like N, Ca, and Zn in grains fluctuated annually, eCO_2_ generally promoted the retention of Fe, Mn, Cu, Zn, and Ca in rice husks, reducing their availability in brown rice (Jiang et al., 2020a), suggesting a need for further investigation into the effects of eCO_2_ on element distribution in ‘flow’ organs such as branches and husks. Additionally, the observed inconsistencies in element allocation under LC treatment result from the complex interactions between photosynthetic activity, nutrient uptake, and transport processes, which can lead to a concentration effect in grains despite unchanged dry matter distribution in panicles. This phenomenon underscores the intricate balance between source and sink dynamics, necessitating further exploration to fully understand the impacts of climatic variations on crop quality and yield.

## Conclusion

Results from FACE experiments conducted over two consecutive rice seasons showed that an increase in atmospheric CO_2_ concentration resulted in a significant increase in rice biomass and NSC concentration of straw. However, the concentrations of mineral elements showed a decreasing trend under eCO_2_, and the response of the stem was more pronounced than that of the leaf and grain. When compared to naturally growing rice, treatments that reduced the source-sink ratio by cutting off leaves significantly decreased rice biomass and straw NSC concentration but increased the element concentrations. From the perspective of source-sink balance, the increased source (eCO_2_) treatment may dilute some mineral elements, while the reduced source (LC) treatment may concentrate them. Increased CO_2_ concentration causes rice to accumulate more mineral elements from the soil, exacerbating soil mineral deficiency risk. Integrating these findings, we consider that future strategies in rice cultivation should focus on improving soil organic matter and fertilizer utilization, in order to mitigate nutrient elements loss under climate change conditions.

## Data Availability

The original contributions presented in the study are included in the article/**Supplementary Material**. Further inquiries can be directed to the corresponding author.
