# Supplementary material for: Impacts of elevated CO2 and partial defoliation on mineral element composition in rice
Source: Front Plant Sci. 2024 Nov 18;15:1450893. doi: 10.3389/fpls.2024.1450893 (PMC11608955; doi:10.3389/fpls.2024.1450893)
Supplement: Supplementary file 1 [file Table1.docx]

Impacts of elevated CO_2_ and defoliation on mineral element composition in rice

Bo Gao^a,b^, Shaowu Hu^a^, Mingyuan Zhou^c^, Liquan Jing^a^, Yunxia Wang^d^, Jianguo Zhu^e^, Xingxing Sun^b^, Kai Wang^b^, Yulong Wang^a^, Lianxin Yang^a,^*

a *Key Laboratory of Crop Genetics and Physiology of Jiangsu Province/Co-Innovation Center for Modern Production Technology of Grain Crops of Jiangsu Province, Yangzhou University, Yangzhou 225009, Jiangsu, China*

b *Jiangsu Coastal Area Institute of Agricultural Sciences, Yancheng 224002, Jiangsu, China*

c *Suzhou Polytechnic Institute of Agriculture, Suzhou 215008, Jiangsu, China*

d *College of Environmental Science and Engineering, Yangzhou University, Yangzhou 225009, Jiangsu, China*

e *State Key Laboratory of Soil and Sustainable Agriculture, Institute of Soil Science, Chinese Academy of Sciences, Nanjing 210008, Jiangsu, China*

* Corresponding author: Email: lxyang@yzu.edu.cn.

**SUPPLEMENTARY MATERIAL**

**Table S1**

Response of elements concentrations in grain to elevated CO_2_ under CK (no leaf cutting) and LC (cutting off top three leaves at heading ) in 2017 and 2018 growing seasons. Data is excerpted from previous reporting (Gao *et al*.2021a).

| Year | Treatment | CO_2_ | N (mg g^-1^) | Ca (mg g^-1^) | K (mg g^-1^) | Mg (mg g^-1^) | P (mg g^-1^) | S (mg g^-1^) | B (mg kg^-1^) | Cu (mg kg^-1^) | Fe (mg kg^-1^) | Mn (mg kg^-1^) | Zn (mg kg^-1^) |
| --- | --- | --- | --- | --- | --- | --- | --- | --- | --- | --- | --- | --- | --- |
| 2017 | CK | AC | 15.9±0.8 | 0.21±0.01 | 2.87±0.03 | 1.43±0.02 | 4.01±0.03 | 1.24±0.01 | 1.60±0.07 | 5.14± 0.46 | 36.8±1.8 | 46.1±2.9 | 30.4±1.92 |
|  |  | EC | 15.9±0.4 | 0.21±0.01 | 3.02±0.02 | 1.41±0.06 | 3.99±0.09 | 1.27±0.03 | 1.83±0.18 | 4.11±0.54 | 35.4±0.1 | 44.1±1.0 | 29.2±1.7 |
|  |  | % Change | -0.2 ns | 1.7 ns | 5.0 * | -1.3 ns | -0.41 ns | 2.0 ns | 14.9 ns | -20.0 ns | -3.8 ns | -4.4 ns | -3.8 ns |
|  | LC | AC | 17.9±0.8 | 0.23±0.02 | 3.17±0.07 | 1.47±0.01 | 4.20±0.06 | 1.34±0.05 | 1.99±0.34 | 5.14±0.73 | 44.2±7.6 | 46.9±5.2 | 34.3±1.1 |
|  |  | EC | 17.2±0.7 | 0.22±0.01 | 3.42±0.08 | 1.47±0.03 | 4.25±0.10 | 1.30±0.03 | 1.65±0.02 | 4.17±0.63 | 39.2±2.8 | 4579±1.7 | 31.2±1.3 |
|  |  | % Change | -3.7 ns | -5.2 ns | 7.8 + | 0.3 ns | 1.2 ns | -2.8 ns | -17.4 ns | -19.0 ns | -11.3 ns | -2.6 ns | -8.9 ns |
| 2018 | CK | AC | 12.9±0.2 | 0.20±0.010 | 3.52±0.10 | 1.87±0.02 | 4.66±0.08 | 1.19±0.01 | 5.45±0.09 | 4.80±0.66 | 30.6±1.2 | 35.7±2.5 | 22.5±0.8 |
|  |  | EC | 12.9±0.4 | 0.20±0.00 | 3.47±0.02 | 1.84±0.04 | 4.66±0.07 | 1.20±0.01 | 5.36±0.07 | 4.97±0.38 | 25.7±1.1 | 37.7±2.9 | 26.2±0.7 |
|  |  | % Change | 0.2 ns | -1.2 ns | -1.5 ns | -1.3 ns | 0.1 ns | 0.4 ns | -1.6 ns | 3.4 ns | -15.9 * | 5.6 ns | 16.1 * |
|  | LC | AC | 15.9±0.7 | 0.19±0.01 | 3.72±0.03 | 1.92±0.04 | 5.15±0.100 | 1.32±0.05 | 5.22±0.18 | 5.01±0.37 | 29.4±3.5 | 35.6±0.7 | 26.8±0.3 |
|  |  | EC | 15.3±0.4 | 0.19±0.00 | 3.68±0.08 | 1.92±0.01 | 5.18±0.06 | 1.30±0.01 | 5.45±0.16 | 5.08±0.65 | 30.2±3.2 | 37.7±1.9 | 28.7±0.9 |
|  |  | % Change | -3.6 ns | 0.5 ns | -1.0 ns | 0.3 ns | 0.5 ns | -1.1 ns | 4.4 ns | 1.4 ns | 2.7 ns | 6.0 ns | 7.0 ns |
| ANOVA results (*P* value) | | | |  |  |  |  |  |  |  |  |  |  |
| CO_2_ | | | 0.457 | 0.627 | **0.096**↑ | 0.774 | 0.787 | 0.815 | 0.950 | 0.287 | 0.294 | 0.908 | 0.704 |
| LC | | | **< 0.001**↑ | 0.468 | **< 0.001**↑ | **0.021**↑ | **< 0.001**↑ | **< 0.001**↑ | 0.874 | 0.818 | 0.152 | 0.754 | **0.002**↑ |
| Year | | | **< 0.001**↓ | **0.003**↓ | **< 0.001**↑ | **< 0.001**↑ | **< 0.001**↑ | 0.101 | **< 0.001**↑ | 0.431 | **0.001**↓ | **< 0.001**↓ | **< 0.001**↓ |
| CO_2_ × LC | | | 0.465 | 0.807 | 0.499 | 0.568 | 0.696 | 0.397 | 0.577 | 0.979 | 0.833 | 0.904 | 0.305 |
| CO_2_ × Year | | | 0.931 | 1.000 | **0.014** | 1.000 | 1.000 | 0.938 | 0.606 | 0.183 | 0.814 | 0.345 | **0.012** |
| LC × Year | | | 0.215 | **0.040** | 0.118 | 0.720 | **0.022** | 0.284 | 0.463 | 0.872 | 0.430 | 0.750 | 0.794 |
| CO_2_ × LC × Year | | | 0.992 | 0.468 | 0.598 | 1.000 | 0.810 | 0.587 | **0.073** | 0.930 | 0.354 | 0.928 | 0.972 |

AC and EC refer to ambient CO_2_ and elevated CO_2_,respectively. CK and LC refer to no leaf cutting and cutting off top three leaves, respectively. Values are means ± standard error (n = 3). Statistically significant effects are indicated as + *P* < 0.1;** *P* < 0.01;* *P* < 0.05; ns, not significant. ↑ and ↓ represent the positive and negative effects of CO_2_ or LC, respectively, or represent increase and decrease in 2018 compared to 2017, respectively.

**Table S2**

Analysis of variance for the concentrations of non-structural carbohydrate (NSC), macroelements, and microelements in rice straw in response to elevated CO_2_, year, leaf-cutting (LC), and organ in 2017 and 2018 growing seasons..

| ANOVA | NSC | Macroelements | | | | | |  | Microelements | | | | |
| --- | --- | --- | --- | --- | --- | --- | --- | --- | --- | --- | --- | --- | --- |
|  |  | N | Ca | K | Mg | P | S |  | B | Cu | Fe | Mn | Zn |
| CO_2_ | **↑ | **↓ | ns | ns | ns | *↑ | *↓ |  | +↓ | ns | *↓ | ns | *↓ |
| Year | **↑ | **↓ | **↓ | **↓ | *↑ | **↓ | **↓ |  | **↑ | **↓ | **↓ | ns | *↑ |
| LC | **↓ | *↓ | ns | ns | **↑ | **↑ | **↑ |  | **↑ | ns | **↑ | ns | *↑ |
| Organ | ** | ** | ** | ** | ** | * | ** |  | ** | ** | ** | ** | ** |
| CO_2_ × Year | * | * | * | ns | ns | + | ns |  | ns | + | ns | * | ns |
| CO_2_ × LC | ** | ns | ns | ns | ns | ns | ns |  | ns | ns | * | ns | ns |
| CO_2_ × Organ | ** | ns | ns | ns | ns | ** | ns |  | * | ** | ** | + | * |
| Year × LC | ns | ** | * | + | + | ns | ns |  | ** | ns | ** | ns | * |
| Year × Organ | ns | ** | ** | ** | * | ** | ** |  | ns | * | ns | ** | ** |
| LC × Organ | ** | ns | ns | ** | ** | ns | * |  | ns | ns | ns | ** | ns |
| CO_2_ × Year × LC | ns | ns | ns | ns | + | ns | + |  | ns | ns | + | ns | ns |
| CO_2_ × Year × Organ | ns | ns | ns | ns | ns | ns | ns |  | ns | ns | ns | ns | ns |
| CO_2_ × LC × Organ | ns | ns | ns | ns | ns | ns | + |  | ns | ns | + | ns | ns |
| Year × LC × Organ | ns | ** | + | ns | ns | ns | ns |  | + | ns | ns | ns | ** |
| CO_2_ × Year × LC × Organ | ns | ns | ns | ns | ns | * | ns |  | ns | ns | * | ns | ns |

Statistically significant effects are indicated as ** *P* < 0.01; * *P* < 0.05; + *P* < 0.1; ns, not significant. ↑ and ↓ represent the positive and negative effects of CO_2_ or LC, respectively, or represent increase and decrease in 2018 compared to 2017, respectively.

**Table S3**

Analysis of variance (ANOVA) results of the effects of elevated CO_2_ and leaf-cutting treatment (LC) on the mineral elements allocations in rice stem, leaf, and grain in 2017 and 2018 growing seasons.

| **Organ** | **ANOVA** | **N** | **Ca** | **K** | **Mg** | **P** | **S** | **B** | **Cu** | **Fe** | **Mn** | **Zn** |
| --- | --- | --- | --- | --- | --- | --- | --- | --- | --- | --- | --- | --- |
| **Stem** | CO_2_ | ns | ns | +↑ | ns | ns | ns | ns | ns | *↓ | *↓ | +↓ |
|  | LC | **↑ | **↑ | **↑ | **↑ | **↑ | **↑ | **↑ | **↑ | **↑ | **↑ | **↑ |
|  | Year | **↑ | ns | **↓ | **↑ | **↓ | **↑ | **↓ | ns | **↑ | **↓ | **↑ |
|  | CO_2_ × LC | ns | ns | ns | ns | ns | ns | ns | ns | + | ns | ns |
|  | CO_2_ × Year | ns | * | ns | ns | ns | ns | ns | ns | * | ns | * |
|  | LC × Year | ns | * | ** | * | ns | + | ns | ns | ns | * | ** |
|  | CO_2_ × LC × Year | ns | ns | ns | + | ns | ns | * | ns | * | ns | ns |
| **Leaf** | CO_2_ | +↓ | ns | +↓ | ns | *↑ | ns | ns | **↑ | **↑ | ns | ns |
|  | LC | **↓ | **↓ | **↓ | **↓ | **↓ | **↓ | **↓ | **↓ | **↓ | **↓ | **↓ |
|  | Year | ns | ns | *↑ | *↓ | **↓ | ns | **↓ | **↓ | *↓ | **↑ | ns |
|  | CO_2_ × LC | ns | ns | ns | ns | + | ns | ns | ns | * | ns | ns |
|  | CO_2_ × Year | ns | ns | ns | ns | ns | ns | * | + | ** | ns | ns |
|  | LC × Year | ns | + | ** | ns | * | ns | ** | ns | ns | * | ** |
|  | CO_2_ × LC × Year | ns | ns | + | ns | ns | ns | ns | ns | + | + | ns |
| **Grain** | CO_2_ | ns | ns | ns | ns | ns | ns | ns | ns | ns | ns | *↑ |
|  | LC | ns | +↑ | **↓ | **↓ | **↓ | **↓ | **↓ | *↓ | **↓ | ns | **↓ |
|  | Year | *↓ | **↓ | **↑ | ns | **↑ | **↓ | **↑ | ns | **↓ | **↓ | **↓ |
|  | CO_2_ × LC | ns | ns | ns | ns | ns | ns | ns | ns | ns | ns | ns |
|  | CO_2_ × Year | + | + | ns | ns | ns | ns | ns | ns | ns | ns | * |
|  | LC × Year | ns | ns | * | ns | ns | ns | + | ns | ns | ns | + |
|  | CO_2_ × LC × Year | ns | ns | ns | ns | ns | + | + | ns | ns | ns | ns |

Statistically significant effects are indicated as + *P* < 0.1;** *P* < 0.01;* *P* < 0.05; ns, not significant. ↑ and ↓ represent the positive and negative effects of CO_2_ or LC, respectively, or represent increase and decrease in 2018 compared to 2017, respectively.
